# Policy Series: ESPO/ Social Research, Policy, and Practice Section Symposium: Connecting Aging Research to Policy: Insights and Strategies for Early-Career Researchers

**DOI:** 10.1093/geroni/igaa057.2382

**Published:** 2020-12-16

**Authors:** Claire Pendergrast, Jennifer May

## Abstract

A wide range of policy issues, from healthcare to transportation to social insurance, influence health and wellbeing for older adults. Gerontologists have the opportunity to get involved with policy at any scale, from the organizational or community level to local, state, or federal policy. This symposium brings together a diverse panel of emerging and established academics to discuss strategies for early career researchers to understand and participate in aging policy activities. Panelists will discuss opportunities for researchers to contribute their expertise to policy discussions, and will share their own experiences and perspectives on participating in the policy process. Specific topics covered will include aging policy internship opportunities for graduate students, academic involvement with advocacy efforts to promote healthcare access to older adults, strategies for designing and conducting impactful and policy research, approaches to collaboration with diverse stakeholders to connect research to policy, and strategies for communicating policy-relevant research findings to general public and policy audiences. This symposium will reflect the SRPP’s strong commitment to connecting research to policy and practice, and will provide early career scholars with strategies to connect their own research to policy in order to inform decision-making and improve health and quality of life for older adults.

